# Reliability and validity of the Veterans Administration Mobility Screening and Solutions Tool

**DOI:** 10.1186/s12913-022-08745-1

**Published:** 2022-11-05

**Authors:** Christine Melillo, Deborah Rugs, Peter Toyinbo, Blake Barrett, Margeaux Chavez, Linda Cowan, Susan Wyatt, Margaret Arnold, Pauline Hilton, Marie Martin, Jill Earwood, Sheila Cox Sullivan

**Affiliations:** 1 Nursing Innovations Center for Evaluation (NICE), Research and Development Service, James A. Haley Veterans’ Hospital and Clinics, Tampa, FL USA; 2 VA Phoenix Healthcare System, Phoenix, AZ USA; 3 CEO, EarlyMobility.com, Plantation, FL USA; 4 Department of Occupational Health and Safety, Veterans Health Administration, Washington DC, USA; 5 VA North Texas Health Care System, Dallas, TX USA; 6 Western North Carolina VA Health Care System, Asheville, NC USA; 7 Office of Nursing Services, Veterans Health Administration, Department of Veterans Affairs, Washington, DC USA

**Keywords:** Patient mobility, Screening, Patient safety, Staff safety

## Abstract

**Objectives:**

The Veterans Administration (VA) Mobility Screening and Solutions Tool (VA MSST) was developed to screen a patient’s safe mobility level ‘in the moment’ and provide clinical decision support related to the use of safe patient handling and mobility (SPHM) equipment. This evidence-based flowchart tool is a common language tool that enables any healthcare worker at any time to accurately measure and communicate patient mobility and transfer equipment needs across disciplines and settings.

**Methods:**

The VA MSST has four levels and differentiates between the need for powered and non-powered equipment depending on the patient’s independence. Subject matter experts wrote scenarios for interrater reliability and validity testing. The initial VA MSST draft iteration was reviewed by 163 VA staff (mostly physical therapists and occupational therapists) amongst simulation scenarios and provided content validity, and additional insight and suggestions. Revisions were made to create the final VA MSST which was evaluated by over 200 healthcare workers from varied disciplines (including medical doctors, advanced practice registered nurses, registered nurses, licensed practical nurses, certified nursing assistants, occupational therapists, physical therapists, speech therapists, radiology and ultrasound technicians, etc.). An instruction video and eighteen scenario videos were embedded in an online survey. The survey intended to demonstrate the interrater reliability and validity (concurrent and construct) of the VA MSST. Over 500 VA staff (raters) received a survey invitation via email.

**Results:**

Raters (*N* = 230) from multiple disciplines and healthcare settings independently screened patient mobility status for each of 18 scenarios using the VA MSST. The raters were diverse in their age and years of experience. The estimated interrater reliability (IRR) for VA MSST was excellent and statistically significant with an estimated Krippendorff’s alpha (ICC (C, k)) of 0.998 [*95% CI*: 0.996–0.999]. Eighty-two percent of raters reported that *overall* VA MSST instructions were clear or very clear and understandable. VA MSST ratings made by technicians and nursing assistants group correlated strongly (r = 0.99, *p* < 0.001) with the ‘gold standard’ (experienced physical therapists), suggesting a high concurrent validity of the tool. The VA MSST significantly discriminated between the different levels of patient mobility required for safe mobilization as intended (each difference, *p* < 0.0001); this suggests a good construct validity.

**Conclusions:**

The VA MSST is an evidence-based flowchart screening and decision support tool that demonstrates excellent interrater reliability across disciplines and settings. VA MSST has strong face and content validity, as well as good concurrent and construct validity.

## Introduction

The Veterans Administration (VA) in partnership with local, Veterans Integrated Service Network 8 Patient Safety Center of Inquiry and Central Office, implemented an evidence-based Safe Patient Handling and Mobility (SPHM) program. SPHM program encourages and supports practices using assistive devices to mobilize patients safely and limit high-risk manual patient handling tasks. The program has been evaluated by researchers at the James A. Haley Veterans’ Hospital and Clinics Research and Development Service in Tampa, FL [1]. The SPHM program is instrumental in the national development and implementation of SPHM innovations such as the The Veterans Administration (VA) Mobility Screening and Solutions Tool (VA MSST). VA leadership support of SPHM is substantiated by 1) peer-reviewed articles that document reduced musculoskeletal injury rates among nurses after adoption of mechanical means to mobilize patients instead of using manual methods [1, 2]; 2) collaborations and products produced with national partners, including the American Nurses Association [3] and the National Institute for Occupational Safety and Health [4]; 3) citations of VA work by the Joint Commission [5] and others; and 4) national media attention [6].

The VA SPHM Program has promoted staff safety working in inpatient settings including rehabilitation, acute care, and community living centers (CLC) through research, education and advocacy. These SPHM efforts resulted in the installation of SPHM technology such as ceiling lifts and the use of mobile powered and non-powered transfer and ambulation devices in VA facilities nationwide. The SPHM program has demonstrated the use of this technology increases the likelihood that patients are moved more frequently. Patients moving more frequently, by definition, decreases immobility and subsequently lowers risk of immobility-related consequences such as pressure ulcers [7]**,** falls, and urinary dysfunction; lower levels of depression; higher engagement in activities [2, 7]; and patients are less combative [8]. The SPHM program literature has been thoroughly reviewed and has been found to have substantial evidence that the program has multiple benefits for patients and staff [9].

Successful SPHM programs need a screening tool to identify ‘in the moment’ mobility limitations, that can be used a staff from a broad range of disciplines. Such screening could assist in triaging patients appropriate for using SPHM equipment. A standard tool for use by multiple disciplines, providing evidence-based practice clinical decision support related to the use of SPHM equipment, and which is appropriate across multiple health care settings, did not exist.

**Fig. 1:**
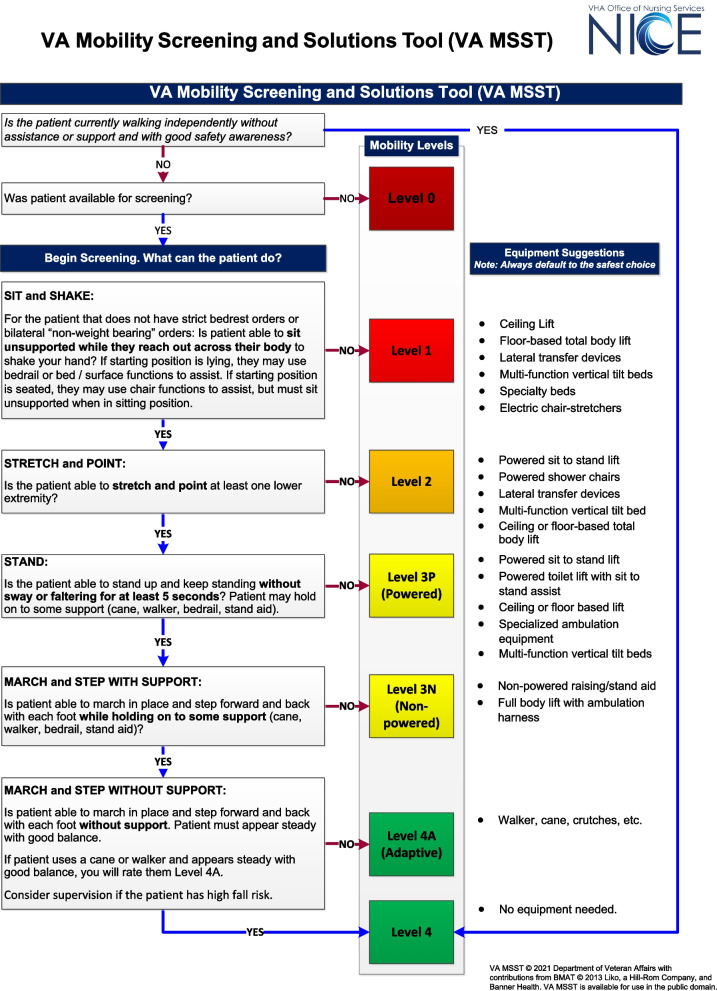
VA Mobility Screening and Solutions Tool (VA MSST)

The VA Mobility Screening and Solutions Tool (VA MSST) (Fig. 1) was developed by revising and enhancing the Bedside Mobility Assessment Tool (BMAT, copyrighted by Banner Health and Hill-Rom, Inc.); a tool designed to be used primarily by nurses to document a mobility assessment. Both BMAT and VA MSST are tools used to describe and document mobility within a patient population. The original BMAT tool showed a good interrater reliability (kappa = 0.91) between three nurses assessing 20 patients [10, 11].

Two commonly used tools to determine patients’ mobility levels, including the Activity Measure for Post-Acute Care short-form ‘6-Clicks’ and BMAT, showed only moderate levels of convergent validity between them, indicating that they do not assess patient mobility similarly [12]. The BMAT, which showed a good interrater reliability (kappa = 0.91) between three nurses assessing 20 patients [10, 11], is used in facilities across the VA. However, it is an assessment tool (versus a screening tool) and designed for nurses within acute care settings. BMAT users across the VA independently identified various gaps which resulted in multiple modified BMAT versions created to address individual scoring and assessment concerns at different VA facilities. At the time of this project, no standard, acceptable tool for mobility screening and solutions existed within the VA.

Revising and enhancing the Bedside Mobility Assessment Tool (BMAT, copyrighted by Banner Health and Hill-Rom, Inc. with their permission) to fill these gaps, we developed the VA Mobility Screening and Solutions Tool (VA MSST) (Fig. 1). The VA MSST is a tool for screening and finding solutions; a pictorial decision flowchart that a caregiver uses to first judge a patient’s mobility status and then match the patient’s mobility status with the best device available. The tool is used to quickly assign the appropriate mobility level (selected from 5 possible levels, including Level 0) to the patient at any given time, acknowledging that mobility levels may change several times even within the same day. As a solution tool each VA MSST level is accompanied with guidance for clinical decisions about which SPHM equipment could be safely used.

Given that raters with varying skill levels will use the VA MSST to rate a diverse patient population with wide range of immobility factors, the VA MSST should produce minimal errors, be equally applicable across patients and demonstrate rater agreement in mobility status assignment. Rating error can lead a rater to determine the inappropriate mobility level of a patient which may result in higher risk of patient falls. The VA MSST also needs to demonstrate strong validity for the intended dual purpose of mobility screening and decision support when choosing SPHM equipment. This manuscript describes the development of the VA MSST tool, its interrater reliability and validity assessments, and qualitative comments from raters on the clarity and ease of use of the tool.

## Methods

Development of the VA MSST was accomplished in the following steps:

1. We reached out to VA facilities through the SPHM program and asked them to share their modifications to the BMAT and rationale for modifications. Nine facilities responded. Modifications varied from adding items to modifying wording. For example, one site added Assessment Level 0 for those who 1) failed Assessment Level 1 (able to sit and shake hands), 2) had myocardial instability or 3) did not respond to voice. These documents, modifications and rationale were then consolidated into one document.
2. An in-person national meeting of VA subject matter experts in SPHM was held June 26–27, 2019. Twenty-two VA SPHM subject matter experts attended in person two-day meeting in Tampa, FL, US. Subject matter experts (SMEs) included physical therapists, occupational therapists, registered nurses, nurse practitioners, anthropologist, industrial hygienist, patient safety and SPHM specialists. Though no current patients were directly involved in this meeting, several of the SMEs had personal experience with both being scored with the tool and using SPHM equipment as a patient. SMEs provided consensus to determine the correct VA MSST score level and equipment needed to safely move a patient within a variety of scenarios. SMEs reviewed the consolidated modifications document from step one (above), various iterations of the VA MSST, identified language and levels that would be improved with change, and made changes to the tool. The tool has five levels (including Level 0) and differentiates between the need for powered and non-powered equipment depending on the patient’s mobility independence.
3. The group of SMEs further developed 18 scenarios containing detailed patient information, (e.g., diagnosis, medication, and reason for the screening, such as “determine appropriate equipment that could allow veteran to stand/walk, transfer and possibly start using hospital bed for sleeping instead of the living room recliner”).
4. The 18 developed scenarios were pilot tested with 161 occupational, physical, and kinesio therapist in a VA simulation lab in August of 2019. Experienced SPHM staff acted out each scenario in a two-day conference. The therapists independently rated the patient (acted by SPHM experts) for their mobility level.
5. Analysis was conducted on the therapist data. Tool education (e.g., training video developed) and scenario suggested changes (e.g., change simulation setting to more accurately simulate car transfer) were addressed. For three levels (MSST Levels 2, 3 N, and 4), new scenarios were developed by the SPHM subject matter experts.
6. Eighteen revised and new scenarios were acted out and filmed at the VA Phoenix medical center, along with an instruction video which were then embedded in an online survey for interrater reliability and concurrent and construct validity evaluation of the final VA MSST Tool.
7. A link to the survey with the videos was emailed to VA staff (independent raters).
8. The final step was the assessment of interrater reliability and validity of the VA MSST. The description of this assessment follows.

### Assessment of interrater reliability and validity of VA MSST

In the summer of 2021, after local Quality Improvement (QI) review/Operations Activity non-research determination and national labor union concurrence, a variety of VA SPHM nursing and rehabilitation email list serves received invitations to participate in an online REDCap survey. The recruitment methods, including snowball sampling the original email list, contained over 2000 VA employee email addresses. Local SPHM facility champions could respond and/or forward the universal survey link to others in their facility. Because individuals may belong to multiple lists serves and snowball sampling was used, we do not have an exact number of staff invited to participate. National SPHM experts produced a seven-minute training video and eighteen brief video scenarios, three for each VA MSST level. All videos were embedded in the survey for training and scoring with the VA MSST. A final sample of participants (*N* = 230) (multiple disciplines and settings) independently rated patient mobility status using the VA MSST and generated the data for VA MSST interrater reliability (IRR) and validity investigation.

### Interrater reliability

Three kinds of rating agreement analyses are often used to evaluate a new rating system including (1) accuracy of agreement, (2) stability of agreement over time, and (3) reproducibility or stability of agreement across current and new sets of independent raters. Of these three, reproducibility (IRR) is the strongest and most feasible to test; the greater the agreement among raters on the data they generate using the VA MSST, the more exchangeable and trustworthy are their data [13]. Krippendorff’s α has been recommended as the standard measure of reliability; its estimation is based on the calculation of disagreement (as opposed to agreement as in most other interrater reliability measures). Also, it was preferable given that it provides stable estimates in the presence of missing data [14]. It should be noted that agreement is only a precursor to true reliability because raters can have excellent agreement on the individual values for a series of random numbers.

We used a fully crossed design for our interrater study such that patient scenarios were rated by the same set of multiple raters, and our ratings comprised ordinal variables. We considered multiple options for quantifying interrater reliability (IRR) for the MSST tool, including Cohen’s [1] weighted kappa, and intra-class correlation (ICC), another commonly used statistic. We chose ICC over kappa and its variants for two reasons: unlike ICC, kappa is more suited to nominal data, and it does not incorporate the magnitude of the disagreement to compute IRR. With ICC, larger magnitude disagreements result in lower ICCs than smaller magnitude [2]. Therefore, using the two-way rater × patient interactions model we estimated intra-class correlation (ICC) statistic for assessing interrater reliability (IRR). We specified average-measures ICCs because all patient scenarios were rated by multiple raters.

Our next challenge was that 22 raters recorded a total of 36 (1.4%) missing values across 14 of 16 patient video scenarios (rows) in our dataset. Although the missing percentage was small, most ICC procedures in R use list-wise deletion for missing data and only 4 rows of data would have been left. We decided to use a less well-known IRR statistic Krippendorff’s α because it provides stable estimates in the presence of missing data [14]. It uses the generalization of the three cases that differentiate the Intraclass correlations (ICCs), and the measure corrects for the raters’ biases [13]. For comparison, we assumed missing at random, performed simple missing data imputation using the method of kNN (k nearest neighbor), and estimated alternative ICC with the imputed data. The R-package irr and irrNA (Coefficients of Interrater Reliability) which allowed for missing data without data imputation were used.

### Validity

After establishing high interrater reliability of the VA MSST, our next step was to assess the tool’s validity; that is, its ability to effectively determine patient mobility level and their SPHM equipment needs as purported. There are different types of validity (although not all are relevant or feasible in the context of the VA MSST as a screening tool for patient mobility level). Therefore, assessment of validity of a tool (or test or measure) is often broad and is tested using both statistical tests and non-statistical methods.

With respect to the VA MSST, content validity reflects the degree to which its content covers known theoretical domains of the target concept or construct, that is, patient mobility level. Content is non-statistical and we assessed it through iterative subject matter expert review and piloting, which ensured optimal coverage and relevance of the contents to the task at hand. The survey also included a question to assess content comprehension in terms of how clear and understandable the instructional contents were.

Secondly, clinical judgement is the basis for making decisions regarding patient mobility level. Therefore, we considered the decision of the experienced physical therapists among the raters as a ‘standard skill’ in clinical judgement (another form of ‘gold standard’). A high correlation between the VA MSST scores generated by experienced physical therapists and those generated by less skilled raters on the same patient scenarios suggests high concurrent validity of the tool. The following professions were included in the less skilled group: certified nursing assistant, dental technician, nursing assistant, occupational therapy aide or technician, patient transporter, physical therapy aide or technician, radiology, or ultrasound technician, and other (see Table 1). We calculated the average VA MSST score per patient scenario to obtain 18 scores (18 scenarios) separately for the skilled versus less skilled and tested correlation between both sets of values.

**Table 1:** Sociodemographic Characteristics of Raters

Characteristic.	*N*	*N* = 230
**Role, n (%)**	228	
Advanced Practice Registered Nurse		21 (9.2%)
Certified Nursing Assistant*		13 (5.7%)
Dental Technician*		4 (1.8%)
Kinesiotherapist		1 (0.4%)
Licensed Practical Nurse		14 (6.1%)
Nursing Assistant*		6 (2.6%)
Occupational Therapist		32 (14%)
Occupational Therapy Aide or Technician		1 (0.4%)
Other*		6 (2.6%)
Patient Transporter*		1 (0.4%)
Physical Therapist		42 (18%)
Physical Therapy Aide or Technician*		2 (0.9%)
Physician		1 (0.4%)
Radiology or Ultrasound Technician*		12 (5.3%)
Registered Nurse		67 (29%)
Speech Therapist		5 (2.2%)
(Missing)		2
**Age group, n (%)**	227	
24 or less		4 (1.8%)
25–34		26 (11%)
35–44		64 (28%)
45–54		69 (30%)
55 or greater		64 (28%)
(Missing)		3
**Years of general experience, n (%)**	228	
(1) < 1		7 (3.1%)
(2) 1 to < 5		12 (5.3%)
(3) 5 to < 10		39 (17%)
(4) 10 to < 20		74 (32%)
(5) 20 to < 30		58 (25%)
(6) 30 or greater		38 (17%)
(Missing)		2
**Years of experience in VA, n (%)**	228	
(1) < 1		25 (11%)
(2) 1 to < 5		56 (25%)
(3) 5 to < 10		66 (29%)
(4) 10 to < 20		62 (27%)
(5) 20 to < 30		16 (7.0%)
(6) 30 or greater		3 (1.3%)
(Missing)		2
**Inpatient setting, n (%)**	230	115 (50%)
**Outpatient setting, n (%)**	230	109 (47%)
**CLC setting, n (%)**	230	49 (21%)
**Rehabilitation setting, n (%)**	230	46 (20%)
**Home setting, n (%)**	230	14 (6.1%)
**Other setting, n (%)**	230	115 (50%)

* These roles were combined to ‘technicians and nursing assistants’ for comparison to physical therapists in Fig. 3

Finally, we expected the VA MSST to discriminate well between levels of mobility when used to select the appropriate equipment for safe mobilization of patients. While the goal here concerns a limited scope of patient mobility level, a significant difference in VA MSST rating score between pairs of mobility levels would suggest a good ‘construct’ validity of the VA MSST. Here we use ‘construct’ in a narrow scope of patient mobility level. To assess construct validity, we performed a non-parametric two-sample Wilcoxon test to compare the distribution of pair vectors of data for adjacent mobility levels.

### Rater’s feedback about user experience of VA MSST compared to the BMAT

Raters compared their experiences with the BMAT, if they previously used that tool, to the VA MSST by answering online REDCap survey questions related to the clarity and understandability of the tool.

### Qualitative responses

Each scenario generated the additional opportunity to leave an open-ended response to a question at the end of the scenario which asked, “Please share any comments on this scenario.” A final open-ended question asked participants to share any other comments they may have on the VA MSST. Open-ended responses were coded for thematic analysis by a qualitative researcher with expertise in survey data analysis. Multiple codes may have been applied to data from a single open-ended response to capture all key issues / emergent themes contained within one open-ended response.

## Results

### Sample description

**Fig. 2:**
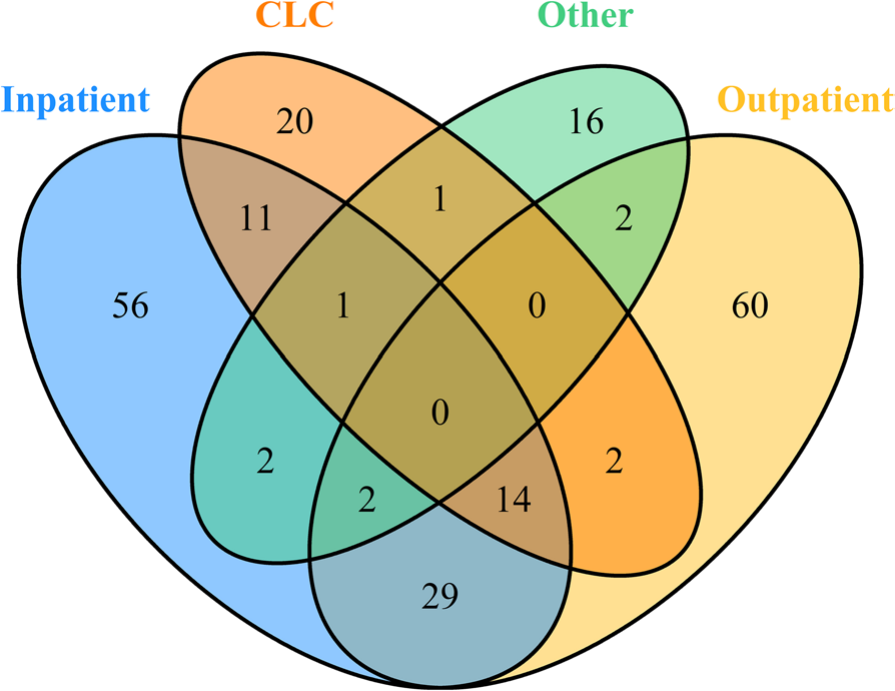
Venn Diagram Showing Overlapping Distribution of VA MSST Raters Across the Top Four Healthcare Settings

Table 1 presents demographics for VA MSST pilot test participants. The raters (*N* = 230) were diverse in their age, professional roles, years of experience, and the health care settings in which they worked. Two-thirds (152/230) of raters reported working in only one type of setting: inpatient (56); outpatient (60); CLC (20); and the remainder [15] work in other settings (Fig. 2). The greatest overlap was found for 45 raters (2 + 14 + 29) who reported working in both inpatient and outpatient settings (Fig. 2).

### Interrater reliability

Overall, the estimated interrater reliability (IRR) for VA MSST was in the excellent range and statistically significant. The Krippendorff’s alpha (ICC(C,k)) was estimated as 0.998 [*95% CI* = 0.996–0.999] *p* < .001, while the imputed data based ICC was 0.996 [*95% CI* = 0.993–0.998], *p* < .001. The high ICCs suggest that raters introduced a minimal amount of measurement error and they had a high degree of agreement. Furthermore, the VA MSST scores demonstrated excellent IRR across settings and disciplines/roles. The approximate mean number of ratings per patient was 229, indicating a good response of participants for each scenario. The representation of inpatient and outpatient settings was very robust (Fig. 2).

### Concurrent and construct validity

**Fig. 3:**
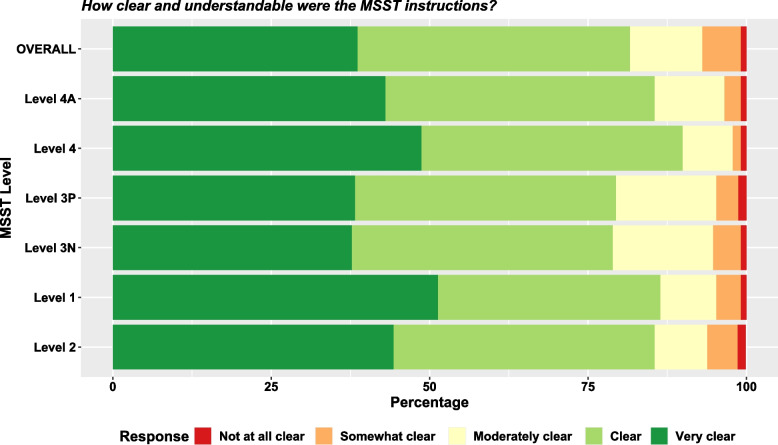
Stacked Bar-Charts of Raters’ Perception of VA MSST Level Instructions

Most raters (82%) reported that *overall* VA MSST instructions were clear or very clear and understandable. More than three quarters reported *every individual level* of VA MSST instruction was clear or very clear and understandable (Fig. 3).

**Fig. 4:**
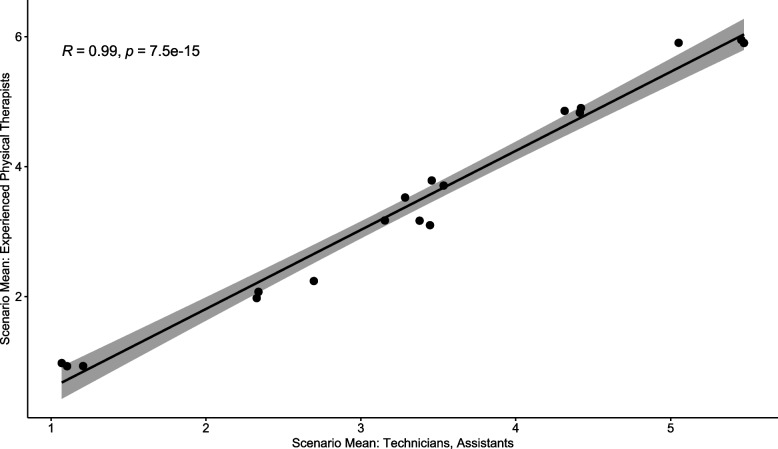
Experienced Physical Therapists Compared to Technicians and Nursing Assistants on Their Average Ratings per Mobility Level

All 42 physical therapist participants, except two, reported at least 5 years of general experience. The latter two therapists reported 1–5 years of experience. The VA MSST ratings provided by experienced physical therapists correlated strongly (*r* = 0.99, *p* < 0.001) with those provided by the less skilled among the raters (i.e., ‘technicians and nursing assistants’) (Fig. 4), suggesting a high concurrent validity coefficient for VA MSST.

**Fig. 5:**
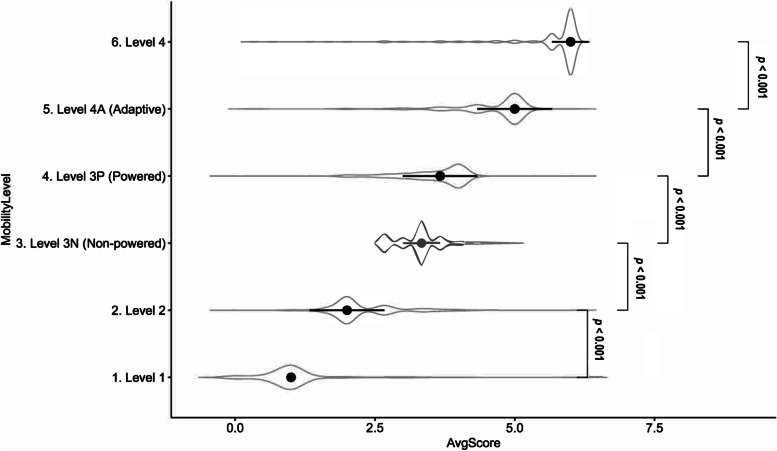
Violin Plots (Error Plots) Showing Median (Point) and Inter-Quarter Range (Bar) of Ratings by MSST Level

The VA MSST shows good discrimination between the different levels of patient mobility required for safe mobilization as intended (Fig. 5). The figure reveals a step increase in summary rating from left to right, and there is significant difference between each pair of adjacent levels (*p* < 0.0001). This suggests that that the VA MSST has good construct validity within the applicable scope of patient mobility concept.

### Comparison to the BMAT

From Table 2, a third of the raters (*n* = 69) reported previous experience with BMAT. Of these, 50 (72%) endorsed VA MSST instructions overall as clear or very clear compared to only 42 (61%) who endorsed BMAT as comfortable or very comfortable to use. The estimated relationship between reported VA MSST clarity of instructions and BMAT comfortability was statistically significant at 95% confidence level, with *p*-value = 0.025.

**Table 2:** Comparison of Raters’ experience with MSST vs. BMAT

		How comfortable using BMAT		
Variable	Overall, *N* = 69^a^	Comfortable/Very, *N* = 42^a^	Not/Some/Moderate, *N* = 27^a^	*p*-value^2^
**MSST instructions: Overall**				0.025
Clear/Very clear	50 / 50 (100%)	35 / 50 (70%)	15 / 50 (30%)	
Not/Some/Moderate	19 / 19 (100%)	7 / 19 (37%)	12 / 19 (63%)	
**(1) MSST instructions: Level 1**				0.014
Clear/Very clear	55 / 55 (100%)	38 / 55 (69%)	17 / 55 (31%)	
Not/Some/Moderate	14 / 14 (100%)	4 / 14 (29%)	10 / 14 (71%)	
**(2) MSST instructions: Level 2**				0.014
Clear/Very clear	55 / 55 (100%)	38 / 55 (69%)	17 / 55 (31%)	
Not/Some/Moderate	14 / 14 (100%)	4 / 14 (29%)	10 / 14 (71%)	
**(4) MSST instructions: Level 3P**				0.028
Clear/Very clear	52 / 52 (100%)	36 / 52 (69%)	16 / 52 (31%)	
Not/Some/Moderate	17 / 17 (100%)	6 / 17 (35%)	11 / 17 (65%)	
**(3) MSST instructions: Level 3 N**				0.039
Clear/Very clear	47 / 47 (100%)	33 / 47 (70%)	14 / 47 (30%)	
Not/Some/Moderate	22 / 22 (100%)	9 / 22 (41%)	13 / 22 (59%)	
**(5) MSST instructions: Level 4A**				0.052
Clear/Very clear	51 / 51 (100%)	35 / 51 (69%)	16 / 51 (31%)	
Not/Some/Moderate	18 / 18 (100%)	7 / 18 (39%)	11 / 18 (61%)	
**(6) MSST instructions: Level 4**				0.008
Clear/Very clear	57 / 57 (100%)	39 / 57 (68%)	18 / 57 (32%)	
Not/Some/Moderate	12 / 12 (100%)	3 / 12 (25%)	9 / 12 (75%)	

^a^Statistics presented: n / N (%)

^2^Statistical tests performed: chi-square test of independence; Fisher’s exact test

Of the 69 raters who reported previous experience with BMAT, 27 (39%) endorsed BMAT as Not- or Somewhat- or Moderately-comfortable to use. However, more than half of same raters (15/27; 56%) endorsed VA MSST instructions as clear or very clear overall.

### Qualitative findings

There were 407 open-ended responses (written-in comments) to the 18 scenarios. Of these 407 open-ended responses, most (*n* = 199) described respondents’ rationale for selecting the VA MSST level for each scenario. Respondents suggested the VA MSST may benefit from including mobility-related precautions at each level or general precautions about how to proceed when patients demonstrate good mobility but may still be at risk for falls (e.g., experiencing dizziness). One respondent wrote the recommended equipment for each VA MSST level may not be available in some settings. They reported this may cause confusion or frustration for staff.

Two responses suggested the VA MSST should contain a brief definition and/or examples of when patients are “unavailable for screening”. They suggest that without further explanation of what “unavailable for screening” means, screeners may go back and forth between Level 0 and Level 1. This concern will be addressed in future VA MSST trainings.

The final open-ended survey question asked, “Is there anything else you would like to share about your experience with the VA MSST?” Fifty-eight (25% of participants) responded, and 34 (59%) of these comments were directly related to the VA MSST tool itself. Most of these comments (22 (65%)) were positive VA MSST comments (e.g., ‘quick and easy to use’). Only 4 (12%) VA MSST comments were negative (e.g., ‘too many levels’), 2 (6%) respondents suggested changes to the tool (e.g., ‘add vital signs’) and 6 (18%) were general use questions (e.g., ‘how often will patient be assessed’) Of the 58 participants who responded to the final question, 28 comments were directly related to the survey platform or delivery (e.g., embedded videos). Just over half of these comments were positive (e.g., ‘all of the scenarios are beautiful’), and just less than half were negative (e.g., ‘videos buffered’).

## Discussion

The current VA MSST has demonstrated strong interrater reliability as a common language tool that allows the full range of direct care providers to accurately measure and communicate patient mobility and SPHM equipment needs across clinical contexts. The estimated interrater reliability (IRR) was in the excellent range at 0.998 [0.996–0.999] or 0.996 [0.993–0.998] with missing data imputation. Ratings provided by experienced physical therapists correlated strongly (r = 0.99, *p* < 0.001) with those provided by the less skilled technicians and nursing assistants suggesting a minimal bias of the tool towards certain professions. The tool shows good discrimination between the different levels of patient mobility required for safe mobilization as intended, supporting good construct validity within the applicable scope of patient mobility concept.

It should be noted, the VA MSST does not replace other mobility assessments, especially those used by occupational or physical therapist in rehabilitation specialties. The VA MSST was intended to be used by any healthcare staff, including technicians to move a patient safely from one location to another or form one position to another. In this regard the VA MSST held up well with a wide variety of staff thus fulfilling the project’s objectives. More importantly, we demonstrated anyone can use the VA MSST suggesting it is feasible that future work may demonstrate beneficial use by family caregivers in the home to mobilize their loved ones. Additionally, unlike most mobility assessment tools, it does not need to be performed by licensed RNs or therapists. This makes the VA MSST unique and novel. Furthermore, the tool is also the first to simplify the use of equipment into discrete categories for clinical decision support.

### Limitations

One limitation of this project was the use of video scenarios instead of face-to-face patient encounters. However, these comprehensive scenarios produced by National SPHM experts accurately presented challenges like the ones the raters will face in real-life situations and covered the VA MSST levels in a balanced way.

While the design approach is limited by not having involved actual patients in the national meeting, our approach effectively addressed other challenges. Our comprehensive scenarios approach became a strength of the project because it reduced variability in the face-to-face encounters and in-person acted-out scenarios. As a quality improvement project which is expected to cause minimal disruption to routine services, it would be a great challenge to assemble such a large number of diverse raters to use the tool on the same set of patients with sufficiently diverse levels of mobility. The video scenario approach added robustness to the VA MSST that would be otherwise impractical. Rizzolo et al. and National League for Nursing [15] concluded “Well-designed and facilitated scenarios, delivered in the controlled environment of the simulation center, can be a reliable and valid tool for evaluating the clinical skills of students.”

Users of mobility assessment tools traditionally designed for use only by nurses independently identified various gaps which have resulted in multiple different versions of modified assessment tools used across the VA system. Addressing these gaps, the new VA Mobility Screening and Solutions Tool (VA MSST) provides one standard screening tool with the highest content validity in the field with respect to patient mobility level screening. With its very high interrater reliability, the new VA MSST is also the tool with the widest known coverage of diverse health caregivers and healthcare settings.

With a sample size of 230 raters spread across all VA facilities, who are diverse in age, professional roles, years of experience, and health care settings in which they worked, the target population of workers are reasonably represented by the raters. The raters ratings can be generalized to a larger population of raters. This suggests a good external validity of the current results.

## Conclusions

The VA MSST demonstrates excellent interrater reliability across disciplines and settings. Raters overwhelmingly found the tool efficient and effective to use for all staff, and reportedly see the Tool as a positive step toward improved SPHM across the VA. VA MSST has strong face and content validity, as well as good concurrent and construct validity. Next steps will be to disseminate our findings within the VA, implementing the tool in the VA’s electronic medical record, and conduct ongoing assessment of implementation and outcomes. Future plans include sharing this tool widely in the public domain to positively impact safe patient handling and mobility throughout the nation.

## Data Availability

The datasets used and/or analyzed during the current study are available from the corresponding author on reasonable request.
